# Improved YOLOv8 Algorithm for Water Surface Object Detection

**DOI:** 10.3390/s24155059

**Published:** 2024-08-05

**Authors:** Jie Wang, Hong Zhao

**Affiliations:** 1 Key Laboratory of Advanced Manufacturing and Automation Technology, Education Department of Guangxi Zhuang Autonomous Region, Guilin University of Technology, Guilin 541006, China; 2120221211@glut.edu.cn; 2 College of Mechanical and Control Engineering, Guilin University of Technology, Guilin 541006, China

**Keywords:** YOLOv8, small water surface object detection, MLCA, SENetV2, SIoU

## Abstract

To address the issues of decreased detection accuracy, false detections, and missed detections caused by scale differences between near and distant targets and environmental factors (such as lighting and water waves) in surface target detection tasks for uncrewed vessels, the YOLOv8-MSS algorithm is proposed to be used to optimize the detection of water surface targets. By adding a small target detection head, the model becomes more sensitive and accurate in recognizing small targets. To reduce noise interference caused by complex water surface environments during the downsampling process in the backbone network, C2f_MLCA is used to enhance the robustness and stability of the model. The lightweight model SENetV2 is employed in the neck component to improve the model’s performance in detecting small targets and its anti-interference capability. The SIoU loss function enhances detection accuracy and bounding box regression precision through shape awareness and geometric information integration. Experiments on the publicly available dataset FloW-Img show that the improved algorithm achieves an mAP@0.5 of 87.9% and an mAP@0.5:0.95 of 47.6%, which are improvements of 5% and 2.6%, respectively, compared to the original model.

## 1. Introduction

Floating waste, as a common pollutant, poses a threat to biodiversity and ecological health. Traditional manual retrieval methods are inefficient and costly, making the use of uncrewed boats for detection and retrieval a highly efficient solution. Consequently, water surface target detection technology has become a focal point in the field of research on uncrewed vessels. Traditional target detection methods require the manual design of feature extractors and classifiers, a process that typically demands the expertise and knowledge of domain experts. Moreover, the representational and generalization capabilities of the feature extractors and classifiers are constrained by the designers’ expertise and experience. In complex scenarios with cluttered backgrounds, deformed or occluded targets, and small target sizes under real water surface conditions, traditional detection methods are prone to false and missed detections. These traditional algorithms are usually tailored to specific target types and scenes, making it difficult to accurately detect new target types and scenes. Currently, deep learning-based algorithms, including Faster R-CNN [1], SSD [2], and the YOLO [3] series, have emerged as leading methods for target detection. These algorithms are specifically designed for classic datasets like MS-COCO [4] and PASCAL VOC [5], where they perform exceptionally well. However, due to the challenges posed by complex environmental interferences such as sunlight and water waves, the vast expanse of water surfaces, and the difficulty in recognizing small targets, the detection performance of these algorithms decreases in practical applications.

To achieve better performance in water surface target detection, many researchers have improved and innovated various deep learning-based target detection algorithms. For instance, Lili Zhang et al. [6] improved Faster R-CNN by integrating different feature layers and optimizing anchor box settings, which enhanced the accuracy of water surface target detection. However, as a two-stage detector, it has a high computational load, leading to slower detection speeds, and rendering it unsuitable for real-time detection applications. Xiangwei Mu et al. [7] optimized SSD by using K-medoids to adjust anchor box scales and aspect ratios, improving detection accuracy on water surface targets. Nonetheless, small targets are often overlooked or submerged in background noise during feature extraction, leading to insufficient detection accuracy. Aofeng Li et al. [8] proposed an improved SSD algorithm that replaces the original model’s backbone network with ResNet-50 and introduces a spatial pyramid pooling structure. This significantly improved small target detection but remained suboptimal under environmental interferences such as lighting and water waves. Yuqing Liu et al. [9] proposed an improved water surface target detection algorithm based on YOLOv3, which enhances detection performance in adverse conditions through data augmentation. While this approach helps the model adapt to diverse environments, it does not significantly improve small target detection accuracy. Zhiguo Zhou et al. [10] proposed the CRB-Net network model, which achieved an mAP of 65.0% on their self-built WSODD dataset. Despite demonstrating some detection capabilities, the overall accuracy was insufficient to meet the high-precision demands of practical applications. Subsequently, C.M.S. Figueiredo et al. [11] tested YOLOv5 on the WSODD dataset, showing that YOLOv5 outperformed CRB-Net in detection accuracy. However, its generalization ability in more complex or diverse water surface environments still requires further validation. Linglong Qi et al. [12] improved the YOLOv7-tiny [13] model, improving the model’s sensitivity to small targets, achieving an mAP of 71.1% on the public water surface dataset FloW-Img [14]. Nevertheless, they did not account for the complex environmental factors of water surfaces, leading to false positives and missed detections under lighting and water wave conditions, indicating the model’s adaptability to environmental changes still needs improvement.

Facing the above challenges, this paper proposes an improved YOLOv8 [3] water surface target detection algorithm. Our contributions are as follows:

(1) Adding a smaller target detection head to the original model improves its ability to detect small targets and addresses missed detection issues.
(2) Adding MLCA to the backbone network to integrate features of different scales enhances the model’s flexibility in detecting small targets at varying distances on the water surface.
(3) Adding the SENetV2 channel attention mechanism to the neck component allows the model to adaptively adjust feature channel importance, enhancing sensitivity and accuracy for small targets, and reducing false and missed detections.
(4) Using the SIoU loss function improves boundary box regression accuracy and reduces background noise interference in small target detection.

The subsequent sections of this paper are structured in the following manner: Section 2 presents the methodology, Section 3 covers the experiments and results analysis, and Section 4 provides a summary and outlook of this study.

## 2. Method

**Figure 1:**
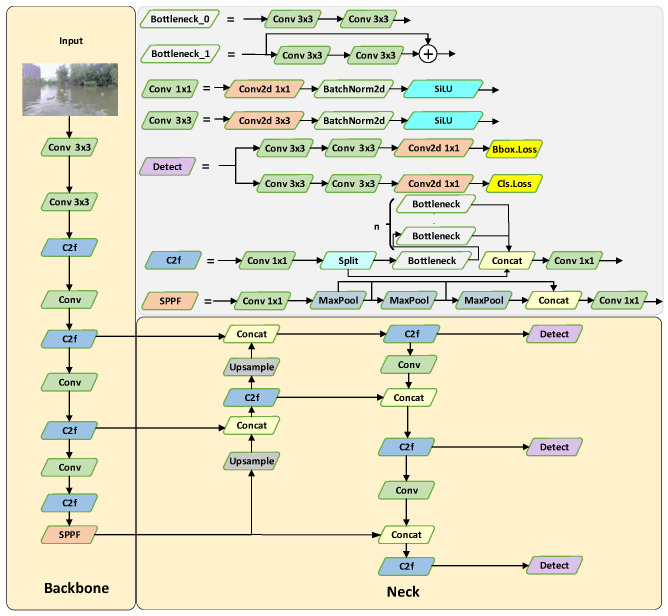
Structure of the YOLOv8.

The YOLOv8 series includes five different network sizes, ranging from smallest to largest: YOLOv8n, YOLOv8s, YOLOv8m, YOLOv8l, and YOLOv8x. These networks share a similar architecture but differ in terms of network size, model depth, the number of channels, and overall performance. In practical applications, larger networks require more memory and computational resources, demanding more expensive hardware to meet real-time requirements. The models within this series are highly portable and modular; a smaller model can be transformed into a larger one by increasing the number of network layers and channels. Conversely, larger models can be developed from the smallest model, YOLOv8n, by reasonably expanding network layers and channels, thereby enhancing the model’s complexity and capabilities. Therefore, this paper focuses on optimizing the smallest and fastest network model, YOLOv8n. In Figure 1, According to the authors’ division of the YOLOv8n network, the architecture primarily comprises three components: backbone, neck, and detection head. The backbone extracts image features, the neck fuses and enhances these features, and the detection head maps these features to the output of the target detection.

### 2.1. Proposed Algorithm

**Figure 2:**
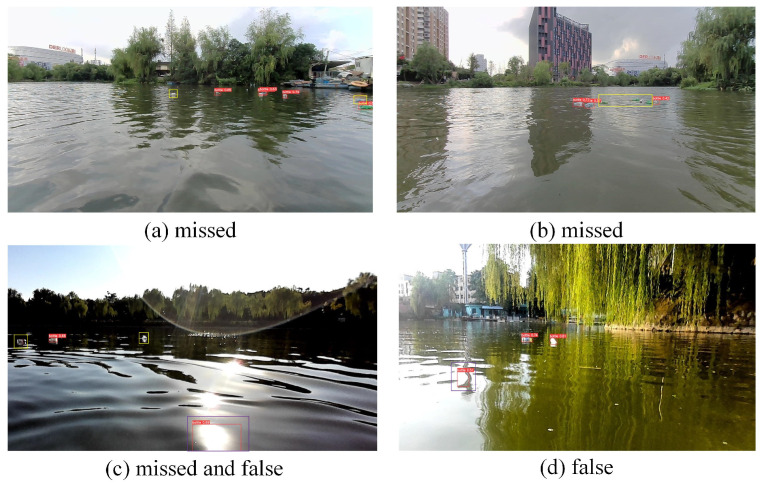
Missed and false detections.

Despite maintaining high detection accuracy at a fast speed, YOLOv8n remains one of the best-performing object detection algorithms. However, when used for detecting floating debris in complex and ever-changing water environments, several issues still arise. In Figure 2, the red rectangles indicate the YOLOv8n detection results, the yellow rectangles show missed detections, and the purple rectangles highlight false detections. In the figure, (a) shows missed detections due to targets being distant and occluded, (b) demonstrates extensive missed detections where small targets are densely clustered, (c) exhibits false detections caused by sunlight reflections on the water surface, as well as missed detections of small, distant targets, and (d) incorrectly detects water waves as targets.

The primary reasons for these issues are as follows:

(1) Targets at different distances appear at varying scales, posing difficulties for the model in performing accurate detection.
(2) Partial or complete occlusion of objects by other elements in the scene can result in missed detections.
(3) Reflections, light variations, and water waves introduce noise that can interfere with detection and lead to false positives.
(4) Small objects, especially when distant or clustered, cannot be reliably detected by the model.

**Figure 3:**
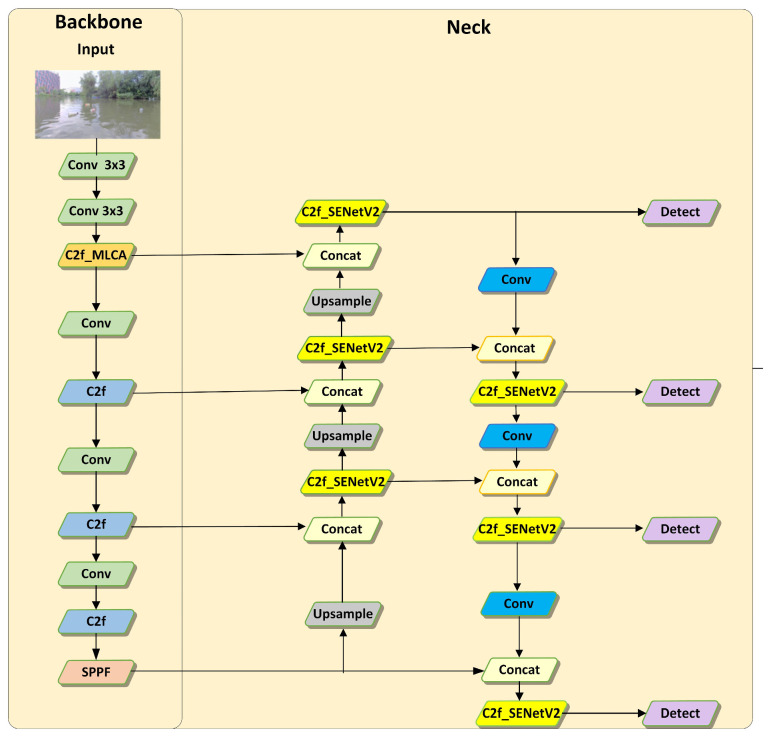
Structure of the improved YOLOv8.

This paper chooses to improve YOLOv8n because it has a low parameter count and fast detection speed, making it an ideal choice for algorithm research. Building on this foundation, we propose the YOLOv8-MSS algorithm. In Figure 3, C2f_MLCA substitutes the C2f in the first layer. MLCA introduces a contextual attention mechanism that better focuses on important regions while suppressing irrelevant background interference, thereby improving the quality of feature representation. MLCA effectively reduces the impact of environmental noise such as lighting variations and water wave reflections on feature extraction, enhancing the model’s robustness and stability. A shallow feature map of size 160 × 160 is obtained through upsampling and feature concatenation to correspond to a smaller detection head. Additionally, C2f_SENetV2 completely replaced the C2f in the neck to enhance the perception of key areas. SENetV2 optimizes feature representation and suppresses noise interference, enabling stable detection performance under various environmental conditions. Moreover, SENetV2 offers higher computational efficiency and lower computational cost, ensuring performance improvements while maintaining the model’s lightweight nature.

### 2.2. Improvements in the Head Component

#### Introducing the MLCA Module

MLCA [15] is a module designed to enhance feature representation and feature fusion. Feature maps at different scales contain varying levels of semantic information. While higher-level feature maps are abundant in semantic content, they exhibit a diminished spatial resolution, while lower-level feature maps offer higher spatial resolution but less semantic detail. By integrating feature maps from various scales, MLCA can more effectively capture the diversity and complexity of targets [16]. In object detection tasks, complex environments often interfere significantly with target recognition. MLCA introduces a contextual attention mechanism that Enhances the model’s concentration on critical regions by considering the background information of the feature maps. This mechanism helps to suppress irrelevant background interference, thereby enhancing the quality of feature representation and detection performance.

First, the input feature map undergoes local average pooling to capture features in localized areas and enhance local information. For example, supposing the input feature map has dimensions of *C* × *W* × *H*, after local average pooling, each block size becomes *ks* × *ks* (where *ks* is the size of the small block), and the output feature dimensions are also modified. Global average pooling (GAP) further compresses the locally pooled feature map into a vector of size *1* × *1* × *C*. The output vector of GAP captures global contextual information, enhancing the representation capability of the feature map. To further extract features and introduce non-linear transformations, MLCA employs a *1D* convolutional layer to process the output of GAP. The output processed by the Sigmoid activation function generates attention weights. During the feature reshaping phase, the output of the convolutional layer is reshaped to match the size of the original feature map. Applying attention weights to the original feature map enhances sensitivity to core features while reducing interference from background information. Finally, the input feature map is combined with the attention weights through element-wise multiplication to produce the final output feature map. This operation enables the model to better locate and recognize targets, which especially enhances its robustness in complex environments.

**Figure 4:**
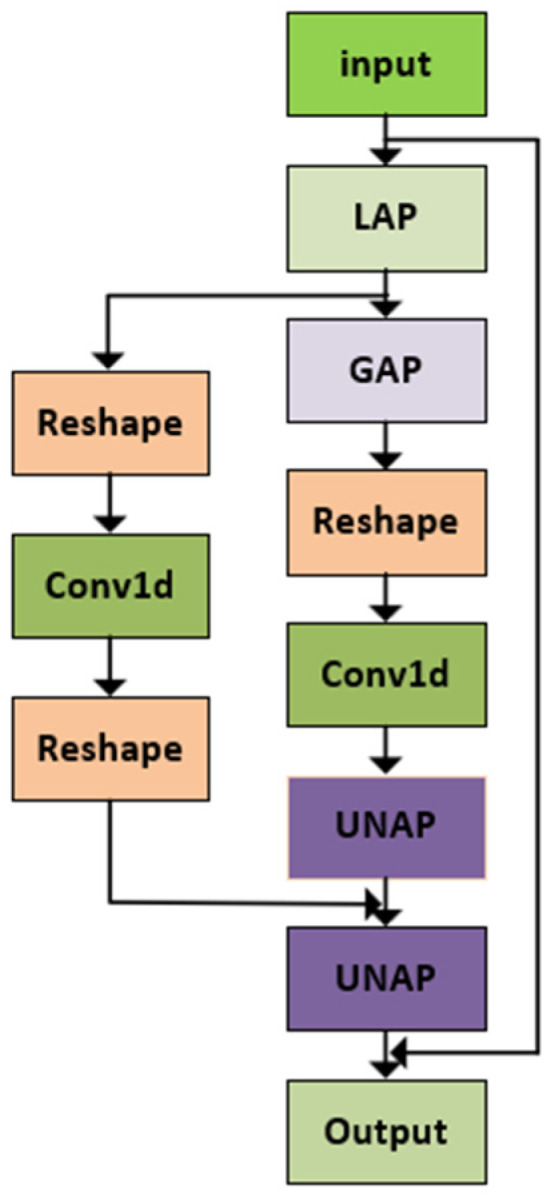
Flow chart of the MLCA module.

Figure 4 illustrates that the Conv1d layer uses one-dimensional convolution, with the kernel size proportional to the channel dimension C, aimed at capturing local features between each channel and its K neighboring channels. The selection of k is determined by Formula (1), which can dynamically determine the appropriate kernel size k, ensuring that the one-dimensional convolution efficiently and effectively captures the interaction information between channels [17]. Adjusting γ and b can control the complexity of the convolution operation to meet the needs of different applications as follows:

(1) $$k=\Phi (C)={\left|\frac{\log_{2}(C)}{\gamma }+\frac{b}{\gamma }\right|}_{\mathrm{odd}}$$

where *C* is the channel number, *k* is the kernel size, and *γ* and odd are hyperparameters, with default values of 2 [15]. If *k* is even, it is incremented by 1.

**Figure 5:**
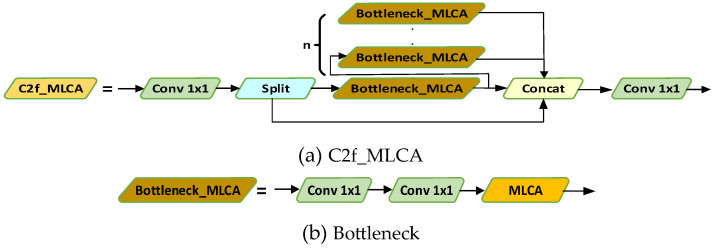
C2f_MLCA module and Bottleneck_MLCA.

Small targets typically take up a minimal part of the image, making them prone to being obscured by background noise. The MLCA module enhances sensitivity to small targets by extracting fine-grained local information through local pooling and then combining it with global pooling to obtain overall context. This multi-level rescaling and fusion of the input feature map enables the model to better distinguish between targets and background, effectively handling occlusions, lighting variations, and background noise in images, thereby improving the stability and reliability of small target detection. C2f_MLCA is utilized to enhance the backbone, as shown in Figure 5.

### 2.3. Improvements in the Neck Component

#### 2.3.1. Adding a Smaller Target Detection Head

In convolutional neural networks, each convolutional layer encompasses a receptive field with a defined extent. With the deepening of the convolutional layers, there is a corresponding reduction in feature map resolution, alongside an expansion of the receptive field for each pixel. This capability allows the network to gather broader global information, but it may also make the detection of small objects more challenging. On the other hand, shallow feature maps have smaller receptive fields and focus more on local information of the original image, providing more useful detail that is often related to small targets [18].

**Figure 6:**
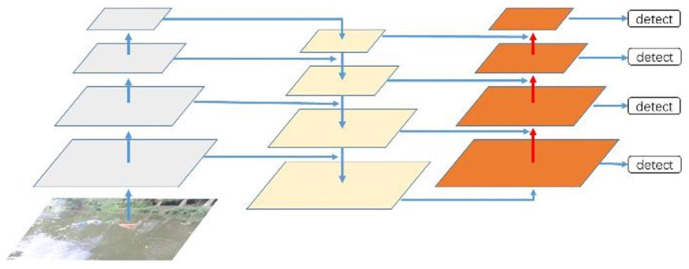
The addition of a smaller detection dead.

YOLOv8n utilizes feature maps at three different scales for object detection. For an input image with dimensions of 640 × 640, the resulting feature maps have sizes of 80 × 80, 40 × 40, and 20 × 20 after being downsampled by factors of 8×, 16×, and 32×, respectively. The YOLOv8n network employs three detection heads on these multi-scale feature maps for object detection. Among these three detection heads, the feature map that results from an 8× downsampling process has the smallest local receptive field. In the scenario of detecting small targets on water surfaces, the features of small targets often blend with the background due to water surface fluctuations and sunlight reflections, making them difficult to detect. Therefore, this paper adds a detection head with 4× downsampling on top of the three existing detection heads. This addition enables clearer capture of smaller target features, enhancing the model’s detection accuracy. By providing finer-grained feature representation, the model can better recognize and distinguish between targets and the background, enhancing its robustness to adapt to different lighting and wave conditions and reducing the missed detection rate. Although adding detection heads increases computational complexity and requires adequate computational resources, lightweight improvements in the model can help reduce this complexity. Therefore, in the application of small target detection on water surfaces, increasing to four detection heads offers significant advantages. The schematic diagram of the added small target detection head is shown in Figure 6.

#### 2.3.2. Introducing the SENetV2 Module

SENet was proposed by Jie Hu et al. [19], with its main innovation being the introduction of the concept of “feature recalibration”. This method aims to enhance the network’s capability to identify key features by evaluating the importance of each channel. SE is a lightweight attention mechanism designed to enhance neural network performance and can be integrated into any layer of a convolutional neural network. The core concept of SENet is to recalibrate the weights of each channel through the SE module, thereby enhancing useful features and suppressing irrelevant ones.

SENetV2 [20] improves and optimizes the original structure by introducing depthwise separable convolutions and other lightweight modules. Based on the idea of “feature recalibration”, SENetV2 improves the process of generating channel weights, allowing for more precise adjustment of each channel’s weight. This further enhances the network’s feature representation capability while significantly reducing computational cost and parameter count. Consequently, SENetV2 can achieve higher performance with the same computational resources. Additionally, SENetV2 incorporates a multi-scale feature fusion mechanism that facilitates the network’s capacity to address targets at multiple scales more adeptly. This improvement is highly effective for images containing targets of varying sizes, significantly enhancing the network’s generalization ability and detection performance.

**Figure 7:**
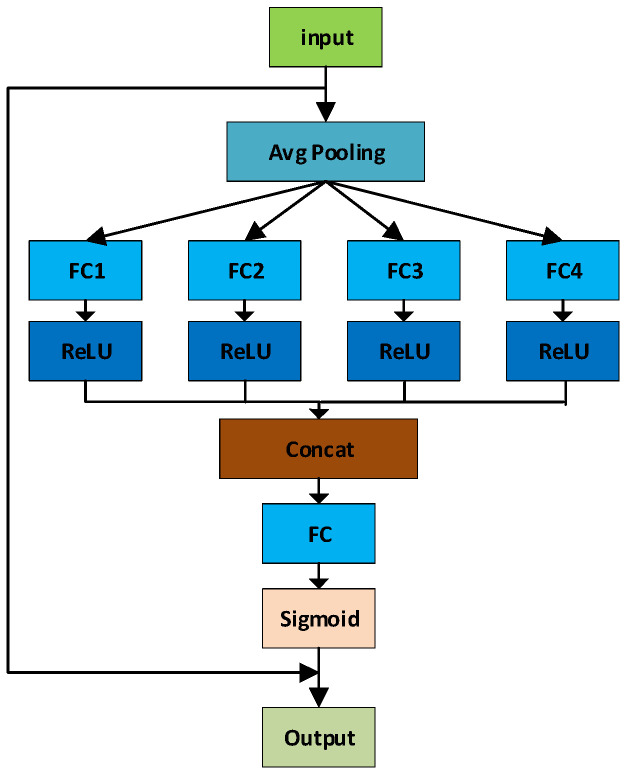
Flow chart of the SENetV2 module.

In Figure 7, SENetV2 utilizes global average pooling (GAP) to compress the spatial dimensions of the input feature map into a single value for each channel, thereby obtaining a global information representation for each channel. This operation captures the overall characteristics of each channel, reducing the influence of spatial dimensions. Specifically, for an input feature map, after applying global average pooling, the global feature for each channel is obtained as follows:

(2) $$z=\frac{1}{H\times W}\sum_{i=1}^{H}\sum_{j=1}^{W}X_{ijk}\;z\in {\mathbb{R}}^{B\times C}$$

Next, SENetV2 introduces four parallel fully connected (FC) layers, where each FC layer performs dimensionality reduction and nonlinear transformation on the input features. This multi-branch design enables the model to capture the diverse and intricate details of the input features, thereby capturing richer feature representations. This is particularly important in water surface target detection, where the environment is complex and variable, requiring the model to have robust feature extraction capabilities to handle different interfering factors. The multi-branch model is represented by the following expression:

(3) fi=ReLU(Wiz+bi)                  fi∈ℝBxcr

where $W_{i}$ and $b_{i}$ are the weights and biases of the parallel fully connected layers, and *r* is the reduction factor.

A ReLU activation function is applied after each fully connected layer, producing various feature representations. This method helps the network to learn diverse features from the input data and thoroughly consider the relationships between different channels. The four reduced-dimension feature representations are then concatenated along the channel dimension to create a new feature vector. This feature concatenation operation integrates rich information from different fully connected layers, enhancing the diversity of the feature representation.

(4) $$F=\mathrm{concat}(f_{1},f_{2},\ldots ,f_{n})\;F\in {\mathbb{R}}^{Bx(n\cdot \frac{c}{r})}$$

Using a fully connected layer combined with a Sigmoid activation function on the concatenated feature vector, this method projects it back onto the initial channel count. The fully connected layer introduces a nonlinear transformation, enhancing the expressive power of the mapping process. This nonlinear transformation can capture more complex feature relationships, contributing to the overall performance of the model. The Sigmoid activation function ensures that the channel weights range from 0 to 1, representing the importance of each channel. This weight-generation mechanism effectively highlights important features and suppresses irrelevant or redundant feature information. This operation enables the model to emphasize crucial information, enhancing the precision of feature selection. The operation is performed using the following expression:

(5) $$s=\sigma (W_{s}F+b_{s})\;s\in {\mathbb{R}}^{BxC}$$

where $W_{s}$ and $b_{s}$ are the weights and biases of the fully connected layer that restores the number of channels.

Finally, the generated channel attention weights are reshaped to B × C × 1 × 1 and multiplied element-wise with the original input feature map to achieve feature recalibration. This channel weighting operation amplifies important feature information, enhancing the expression of critical features while suppressing unimportant ones. This improves the model’s feature selection capability and robustness. The channel weighting operation is performed using the following expression:

(6) $$X\prime =X⊙s\;X\prime \in {\mathbb{R}}^{BxCxHxW}$$

where ⊙ is represented by element-wise multiplication.

**Figure 8:**
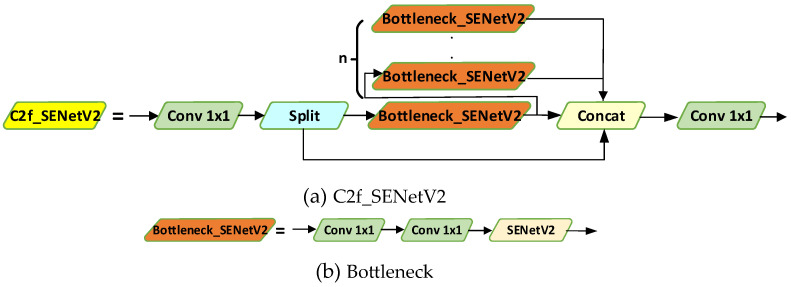
C2f_SENetV2 module and Bottleneck_SENetV2.

In practical water surface target detection environments, numerous complex factors such as lighting changes, water surface ripples, reflections, and vegetation may interfere with target detection. Using SENetV2 can effectively extract regions in the image that are related to the target while ignoring areas that contain only background features. In this way, SENetV2 helps the model focus on useful feature information and ignore irrelevant information from the environment, thus increasing detection accuracy and reliability amidst various interferences. In this paper, C2f_SENetV2 is used to improve the neck, as illustrated in Figure 8.

### 2.4. Loss Function Improvement

The CIoU loss function used by YOLOv8 [21] integrates considerations of IoU, center point distance, and aspect ratio, aiming to enhance the precision of bounding box localization and shape matching. CIoU considers three factors: IoU, the distance between the center points, and the aspect ratio. It seeks to enhance the precision of bounding box localization and shape matching. CIoU is represented by the following expression:

(7) $$\left\{\begin{array}{l} L_{\mathrm{CIoU}}=1-IoU+\frac{\rho^{2}(B_{prd},B_{gt})}{c^{2}}+\alpha \nu \\ \nu =\frac{4}{\pi_{2}}{\left(\arctan\frac{w_{gt}}{h_{\mathrm{gt}}}-\arctan\frac{w}{h}\right)}^{2}\\ \alpha =\frac{\nu }{(1-IoU)+\nu } \end{array}\right.$$

where $\rho (B_{prd},B_{gt})$ represents the Euclidean distance between the center points of the predicted and ground truth boxes, c represents the diagonal length of the smallest enclosing box covering the predicted and ground truth boxes, α is a weight balancing parameter, and ν is the aspect ratio penalty term.

However, when the aspect ratio of the predicted and ground truth boxes is linearly related, the aspect ratio penalty term of CIoU may degrade to zero, resulting in an inability to effectively distinguish between the two [16]. The degradation may occur even with significant differences, failing to provide effective regression guidance. Floating objects on the water surface can tilt and rotate at various angles due to the movement of the water, and CIoU does not account for angular differences, making it inadequate for aligning rotated targets. This limitation results in poor performance when detecting tilted and rotated objects. Additionally, water surface targets are typically small in scale, and CIoU cannot accurately reflect subtle differences between bounding boxes, leading to poor detection performance for small targets.

To improve the model’s localization accuracy, the CIoU loss is replaced by the SIoU loss [22]. SIoU ensures precise spatial alignment between the predicted and ground truth boxes through the center point distance penalty term. This is particularly effective for small targets and subtle positional differences, significantly improving localization accuracy. In complex water surface scenarios, the addition of shape awareness capability enables the model to effectively capture the geometric features of the target, maintaining high robustness and reducing missed and false detections.

SIoU redefines the penalty standards, including the following components:

1. The calculation of the angle penalty is divided into two cases as follows:

When $\alpha <\frac{\pi }{4}$:

(8) $$\Lambda =\cos(2\alpha -\frac{\pi }{2})$$

When $\alpha <\frac{\pi }{4}$:

(9) $$\Lambda =\cos(2\alpha -\frac{\pi }{2})$$

(10) $$\left\{\begin{array}{l} \alpha =\arcsin\left(\frac{C_{h}}{\sigma }\right)\\ C_{h}=\max\left(b_{y}^{gt},b_{y}\right)-\min\left(b_{y}^{gt},b_{y}\right)\\ \sigma =\sqrt{{\left(b_{c_{x}}^{gt}-b_{c_{x}}\right)}^{2}+{\left(b_{c_{y}}^{gt}-b_{c_{y}}\right)}^{2}}\\ \alpha +\beta =\frac{\pi }{2} \end{array}\right.$$

**Figure 9:**
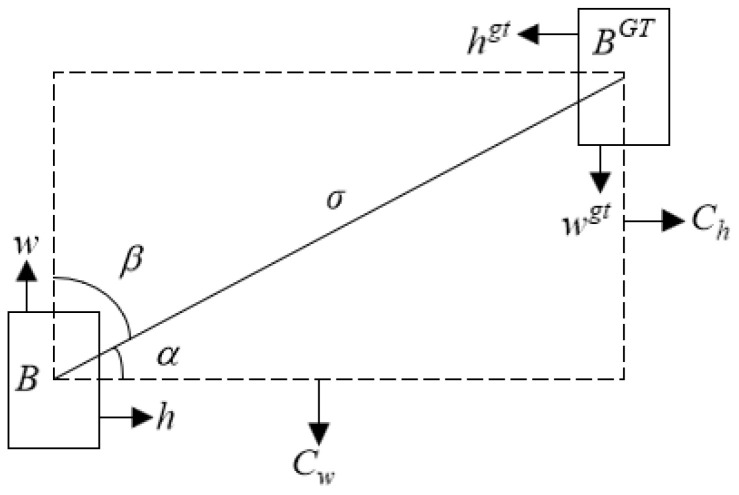
Angle penalty.

In Figure 9, the rectangle B represents the predicted box and $B^{gt}$ represents the ground truth box. Their center coordinates are $\left(b_{x},b_{y}\right)$ and $\left(b_{x}^{gt},b_{y}^{gt}\right)$, and their heights and widths are h,w and $h^{gt},w^{gt}$, respectively. The vertical distance between the center points of the predicted and ground truth boxes is denoted as $C_{w}$, while σ represents the horizontal distance between these center points. $\arcsin\left(\frac{C_{h}}{\sigma }\right)$ represents the angle between the line segment formed by the center coordinates of the ground truth box and the predicted box and the horizontal line.

2. The calculation of the distance penalty is as follows:

(11) $$\left\{\begin{array}{l} \Delta =\sum_{t=x,y}\left(1-e^{-\gamma \rho_{t}}\right)\\ \rho_{x}={\left(\frac{C_{w}}{C_{W}}\right)}^{2},\rho_{y}={\left(\frac{C_{w}}{C_{W}}\right)}^{2},\;C_{w}=b_{x}^{gt}-b_{x},C_{h}=b_{y}^{gt}-b_{y} \end{array}\right.$$

**Figure 10:**
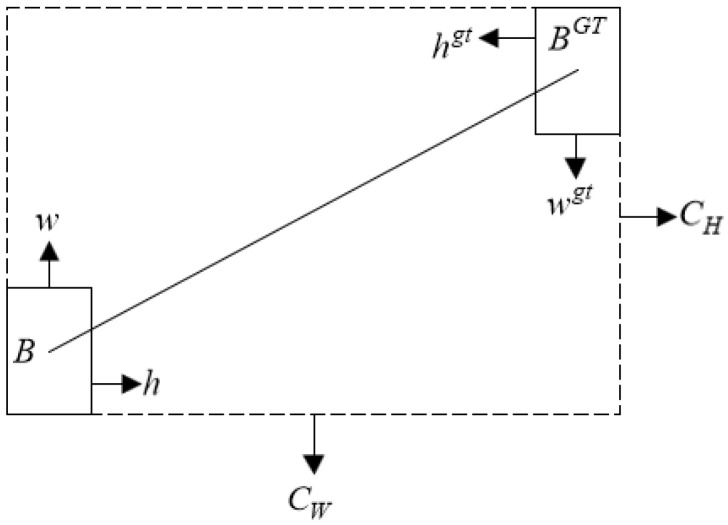
Distance penalty.

In Figure 10, where $C_{H}$ and $C_{W}$ are the height and width of the smallest enclosing rectangle of the predicted and ground truth boxes, respectively, $\rho_{x}$ and $\rho_{y}$ represent the degree of deviation between the positions of the predicted and ground truth boxes, and γ is the distance value assigned with time priority, γ=2−Λ.

**Figure 11:**
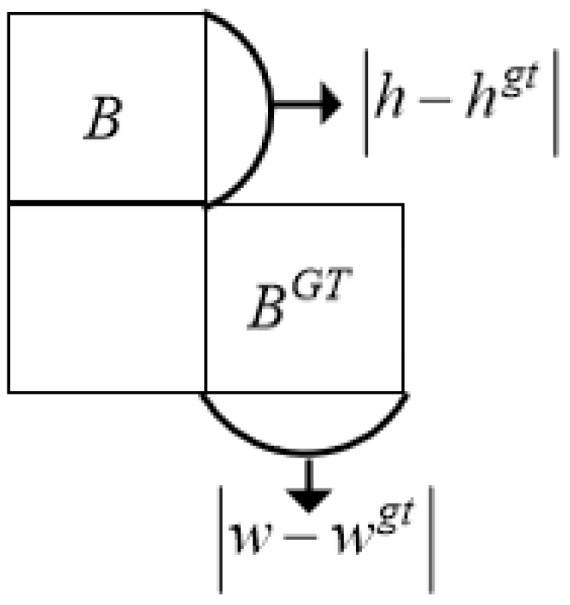
Shape penalty.

3. The calculation of the shape penalty is as follows:

(12) $$\left\{\begin{array}{l} \Omega =\sum_{t=w,h}{\left(1-e^{-\omega_{t}}\right)}^{\theta }\\ \omega_{w}=\frac{\left|w-w^{gt}\right|}{\max\left(w,w^{gt}\right)},\omega_{h}=\frac{\left|h-h^{gt}\right|}{\max\left(h,h^{gt}\right)} \end{array}\right.$$

where θ represents the focus on shape loss. Zhora [22] determined the optimal value using a genetic algorithm, which is close to 4, with a range from 2 to 6. In this paper, θ is set to 4. The shape penalty is shown in Figure 11.

Ultimately, the SIoU loss function is obtained. Compared to CIoU, SIoU accelerates network convergence and reduces the loss function value by focusing on the directionality between the predicted box and the ground truth box ($L_{box}$) as follows:

(13) $$\left\{\begin{array}{l} L_{box}=1-IoU+\frac{\Delta +\Omega }{2}\\ IoU=\frac{\left|b\cap b^{gt}\right|}{\left|b\cup b^{gt}\right|} \end{array}\right.$$

where $\left|b\cap b^{gt}\right|$ represents the area of the intersection between B and $B^{gt}$, and $\left|b\cup b^{gt}\right|$ represents the area of the union of B and $B^{gt}$.

## 3. Experiments and Results Analysis

### 3.1. Experimental Dataset

**Figure 12:**
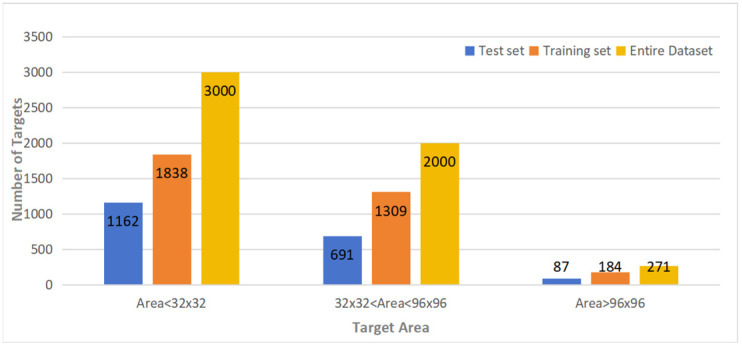
The distribution of the occupied area of the labeled objects.

This study used the publicly available FloW-Img water surface garbage dataset, which is the world’s first dataset for monitoring water surface debris from the perspective of an uncrewed vessel. This dataset includes 2000 images with 5271 annotated water surface targets, mostly comprising common floating garbage such as plastic bottles, cans, and glass bottles, all categorized into a single class. The dataset was allocated into training and testing components, adhering to a 7:3 distribution, allocating 1400 images for training and 600 images for testing. Figure 12 illustrates the distribution of the area covered by annotated objects across the entire dataset, the training set, and the testing set. The figure shows that small target objects (area < 32 × 32) represent the majority. Therefore, the chosen dataset is well suited for evaluating the model’s performance in detecting small targets.

### 3.2. Evaluation Metrics

In this paper, mAP (mean Average Precision) is used as the model detection performance evaluation index. To calculate mAP, the average precision (AP) must first be computed, representing the mean precision for a category within the dataset. The calculation process is as follows:

(14) $$AP=\int_{0}^{1}p(r)d(r)$$

(15) $$mAP=\frac{1}{N}\sum_{i=1}^{N}AP_{i}$$

where $P_{i}$ denotes precision, which is the percentage of correct detections made by the model relative to all detections made by the model; *r* signifies recall, which is the percentage of correct detections relative to all required detections in the dataset; and *N* represents the number of categories in the dataset.

In object detection, FLOPs are used as a metric to measure the computational complexity and efficiency of a model. FLOPs help evaluate the real-time performance and applicability of a model. During model optimization and improvement, FLOPs can also serve as an important evaluation criterion. The FLOPS expression is as follows:

(16) $$FLOPs=2\cdot H\cdot W\cdot C_{in}\cdot K\cdot K\cdot C_{out}$$

where $C_{in}$ denotes the number of channels in the input feature map of the convolutional layer, $C_{out}$ indicates the number of channels in the output feature map, and *K* represents the size of the convolutional kernel.

### 3.3. Experimental Parameters and Environment Configuration

Table 1 provides detailed information on the key parameters of the experiments.

### 3.4. Results Analysis

#### 3.4.1. Backbone Network Improvement Experiment

The original YOLOv8n model’s backbone network contains four C2f modules. To identify the optimal backbone network configuration, the proposed C2f_MLCA modules were incrementally added, and the best-performing configuration was selected through experimentation. As shown in Table 2, MLCA, being a lightweight module, enhances efficient feature selection without significantly increasing the computational load. Incorporating this module can improve the mAP@0.5 by up to 2.2% and mAP@0.5:0.95 by 1.5% compared to the original model. This enhancement demonstrates the effectiveness of the C2f_MLCA module in improving model performance, particularly in terms of accuracy and robustness, without substantially increasing computational requirements. Based on the experimental results, the first C2f module in the YOLOv8n model’s backbone network was replaced with the C2f_MLCA module.

#### 3.4.2. Neck Network Improvement Experiment

In Table 3, adding a smaller object detection head to the original model significantly improves detection accuracy, with mAP@0.5 increasing by 3.9% and mAP@0.5:0.95 increasing by 1.9%. Although there is an increase in GFLOPS, this sacrifice is justified by the substantial gain in accuracy. Furthermore, incorporating the lightweight attention mechanism SENetV2 on top of this results in an additional improvement in model accuracy, with mAP@0.5 improving by 4.2% and mAP@0.5:0.95 improving by 2.1% compared to the original model.

#### 3.4.3. Loss Function Improvement Experiments

**Figure 13:**
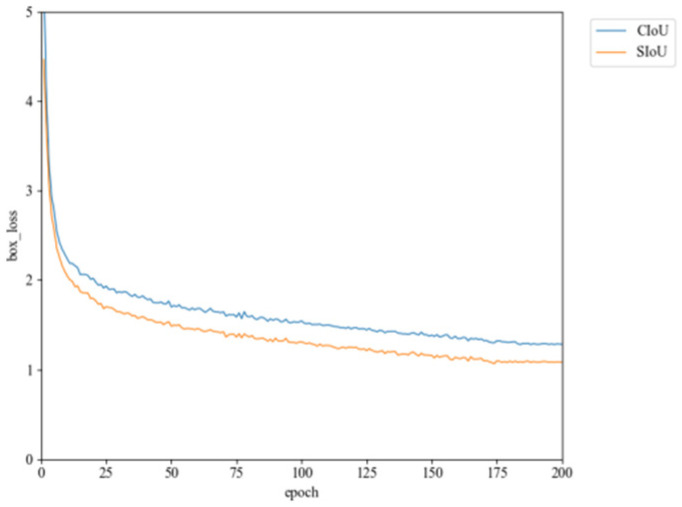
Comparison of loss function.

Comparative experiments were conducted to evaluate the SIoU loss function against other common loss functions such as DIoU [23], WIoU [24], and ShapeIoU [25]. Table 4 indicates that using the SIoU loss function in the YOLOv8n model increases mAP@0.5 by 1.2% and mAP@0.5:0.95 by 1%. The experiments illustrate that using SIoU can control the convergence effect of the prediction frame more effectively and can lead to improved accuracy. Figure 13 shows a comparison of the loss functions used by the original and improved models. It was observed that SIoU converges faster and achieves a lower final convergence value, demonstrating better performance. Therefore, SIoU is used to enhance the model in this paper, aiming to improve training performance and accuracy.

### 3.5. Ablation Experiments

As shown in Experiments 1–5 in Table 5, adding a dedicated smaller target detection head significantly enhances the model’s ability to perceive small targets. Compared to YOLOv8n, the improvements are 3.6% in mAP@0.5 and 1.9% in mAP@0.5:0.95. Although the GFLOPs increased by 4.1% compared to the baseline model, this trade-off is justified by the significant improvement in accuracy. Experiments 7, 8, 11, and 14 indicate that without the smaller target detection head, it is difficult to achieve significant improvements in detection accuracy. This underscores the necessity of the small target detection head as a foundational enhancement. On this basis, the addition of MLCA, SENetV2, and SIoU (in experiments 6, 9, and 10) resulted in mAP@0.5 improvements of 3.9%, 4.2%, and 4.1%, respectively. Combining C2f_MLCA, Smallerhead, C2f_SENetV2, and SIoU led to the most significant performance improvement, demonstrating the complementary nature of these modules in enhancing detection accuracy and robustness. When SIoU loss function was added in experiments 13 and 15 (on top of experiments 6 and 9), additional mAP@0.5 improvements of 0.5% and 0.4% were achieved, respectively. This is because the SIoU loss function better considers the positional and scale variations in the target boxes, thus excelling in small target detection tasks.

After 200 iterations of training, the YOLOv8-MSS model, which incorporates MLCA, SENetV2, Smallerhead, and SIoU, improved mAP@0.5 by 5% and mAP@0.5:0.95 by 2.6% compared to YOLOv8n. The improved model demonstrates higher accuracy in target detection tasks, effectively identifying and locating targets. It also performs more stably across different IoU thresholds, allowing for finer adjustments of the predicted boxes and improving boundary box regression accuracy. The YOLOv8-MSS model exhibits superior performance in complex environments and small target detection tasks, validating the effectiveness and necessity of these enhancements.

### 3.6. Comparative Experiments of Different Models

#### 3.6.1. Comparison of Common YOLO Series

In order to demonstrate the superiority and effectiveness of the improved algorithm model proposed in this paper, the same dataset is used to compare with the YOLO series of algorithms under the same experimental conditions. YOLOv5s utilizes the Focus structure to enhance the receptive field and optimize anchor boxes, making their sizes more suitable for small targets in specific datasets, thereby improving the detection accuracy of small targets. YOLOv6 [26] enhances the Feature Pyramid Network and Path Aggregation Network for more efficient multi-scale feature fusion, thereby improving the detection capability for small targets. YOLOv7 utilizes the efficient ELAN network architecture to improve the detection of small targets.

**Figure 14:**
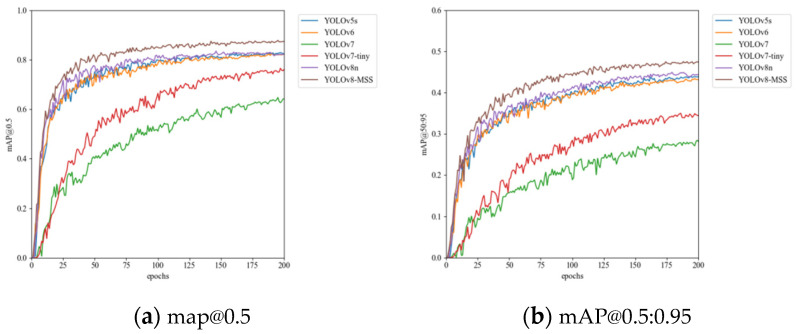
mAP curve comparison of different models.

In Table 6, the improved YOLOv8-MSS model outperforms other models in both mAP@0.5 and mAP@0.5:0.95. The YOLOv9 [27] adopts a more advanced backbone network, which further improves feature extraction capability. Although it significantly enhances accuracy, the high computational complexity of 102.3 GFLOPs greatly reduces the inference speed of the model. Although the YOLOv8-MSS model is 0.8 M larger than YOLOv5s, its accuracy is far superior. In terms of F1 scores, YOLOv8-MSS is higher than all other models. The experimental results indicate that YOLOv8-MSS offers notable benefits in tackling small object detection challenges, as illustrated in Figure 14.

**Figure 15:**
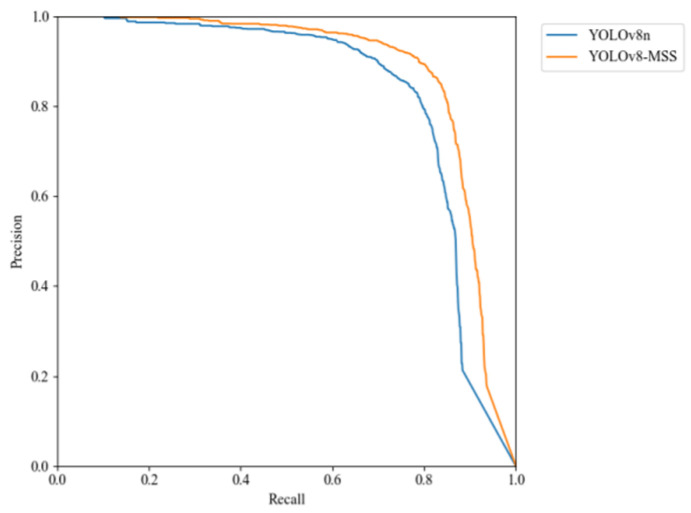
Comparison of P-R curves.

Figure 15 compares the Precision–Recall (P-R) curves of the model before and after improvements. If the P-R curve of one learner is entirely within the P-R curve of another learner, it indicates that the latter exhibits superior performance [28]. In the figure, it is clearly shown that the P-R curve of the YOLOv8-MSS model completely envelops the P-R curve of the original model, indicating that the overall performance of the improved model has been enhanced.

#### 3.6.2. Comparison of Other Algorithms

To compare the effectiveness of the improved algorithm presented in this paper with other algorithms, it was evaluated against Faster R-CNN, SSD, Rt-detr [29], and other enhanced models using the FloW-Img dataset. In Table 7, YOLOv8-MSS achieved improvements of 31.7%, 9.9%, and 17.3% in mAP@0.5 compared to Faster R-CNN, SSD, and Rt-detr, respectively, demonstrating significant gains and also having the smallest model size. YOLO-Float [30] combines a low-level representation enhancement module with an attentional fusion module to improve sensitivity to small targets and model robustness. YOLOv7-CA Dynamic [31] employs the concepts of feature separation and merging, incorporating attentional mechanisms and dynamic convolution to reduce the impact of feature loss and noise. The improved models offer higher accuracy and are considerably smaller in size compared to YOLO-Float and YOLOv7-CA Dynamic, making them more difficult to deploy on resource-limited surface robots.

### 3.7. Visualization Analysis

**Figure 16:**
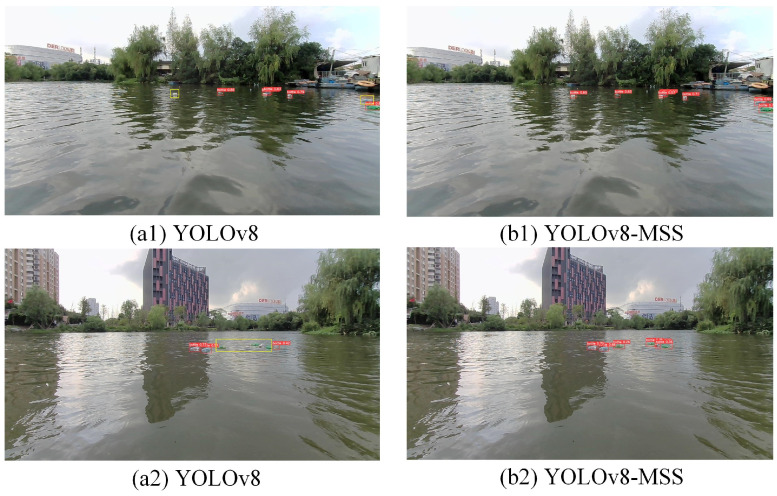
Comparison of small target detection performance.

**Figure 17:**
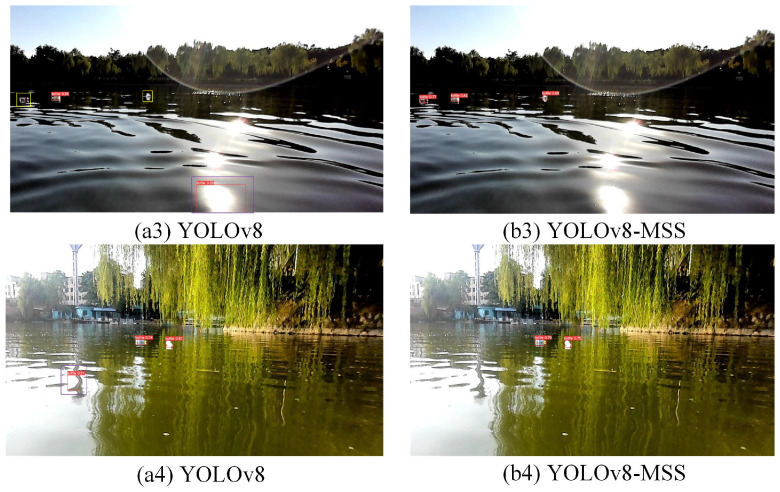
Comparison of anti-interference capabilities.

To visually illustrate the effectiveness and superiority of YOLOv8-MSS, some images were selected for testing with both the proposed model and the YOLOv8n. The detection results are shown in Figure 16(a1)–Figure 17(b4), where Figure 16 and Figure 17(a3,a4) represent the YOLOv8n model test results, and Figure 16 and Figure 17(b3,b4) represent the YOLOv8-MSS model test results.

In Figure 16(a1), it can be clearly seen that the original model has a weak ability to detect small targets, leading to missed detections. In Figure 16(a2), the objects to be detected are relatively dense, and most of them are small in size, with some being occluded, which significantly affects the detection performance of the original model. Figure 16(b1,b2) clearly show that the YOLOv8-MSS can more effectively detect all small target garbage in the images.

In Figure 17(a3,a4), it can be seen that reflective and wave interference on the water surface causes the original network model to produce false detections, and small distant targets are also missed. In comparison, Figure 17(b3,b4) demonstrate that the YOLOv8-MSS model has strong anti-interference capabilities and performs better in detecting small targets.

### 3.8. Generalisation Experiment

To evaluate the model’s generalization ability, experiments were conducted on the WSODD [11] dataset. This dataset comprises 7467 images captured by high-definition cameras under varying water surface environments, times of day, and weather conditions, and is categorized into 14 object classes. Among these images, there are 11,564 instances of small targets, which make up 53% of the total instances, reflecting a realistic water surface environment dominated by small targets and presenting a significant challenge for detection.

In Table 8, the detection performance for larger and medium-sized objects such as bridges, ships, boats, and rocks is comparable between the models. However, significant improvements were observed in the detection of smaller targets such as small animals on the water surface, distant people, balls, and floating glass, with enhancements of 8%, 10.7%, 8.7%, and 7.8%, respectively. Additionally, the improved model showed a 3.1% increase in mAP@0.5 compared to YOLOv8n.

To further evaluate the effectiveness of the YOLOv8-MSS, its effectiveness in detecting small targets was tested on objects outside of water surfaces. The VisDrone2019 dataset, collected by the Tianjin University laboratory team using drones across various cities in China under different scenarios, weather conditions, and lighting, was utilized. This dataset includes 10 object categories and features common targets in transportation, making it a standard dataset for evaluating small target detection.

In Table 9, the detection performance for all object categories has improved to varying degrees. The model demonstrated enhanced capability in extracting features for small vehicles such as bicycles, tricycles, and motorcycles, in the detection of pedestrians, people, and motor vehicles, the mAP@0.5 achieved improvements of 8.2%, 7.9%, and 6.6% respectively, leading to an overall average precision increase of 4.2%, thereby validating the effectiveness of the improved model in enhancing small target recognition.

## 4. Conclusions

In water surface target detection, small targets are challenging due to their limited pixel coverage in images, which provides minimal information and often leads to missed detections, resulting in lower accuracy. Additionally, the detection performance is adversely affected by environmental factors such as lighting and water waves, leading to false detections. To address these issues, this paper proposes the YOLOv8-MSS object detection algorithm. This approach introduces a small target detection head to the original model, making it more sensitive to small targets. In the backbone component, C2f_MLCA is used to reduce noise interference during downsampling in complex water environments. In the neck component, C2f_SENetV2 replaces the original C2f to enhance detection and robustness. SIoU is employed instead of CIoU as it better considers the geometric attributes of bounding boxes, reducing localization errors and improving detection performance. The effectiveness of the improved model was validated using the publicly available FloW-Img dataset, achieving mAP@0.5 and mAP@0.5:0.95 of 87.9% and 47.6%, respectively, with improvements of 5% and 2.6% over the original model. Generalization experiments on other datasets further validate the model’s small target detection capability and robustness against interference. Future work will focus on expanding the dataset to include data from special conditions such as rain, fog, snow, and nighttime to enhance model robustness. Additionally, efforts will be made to reduce computational resources while maintaining high detection accuracy to facilitate deployment on resource-constrained devices.

## Figures and Tables

**Table 1:** Experimental key parameters.

Training parameters	Optimizer	SGD
	Batch size	8
	Epochs	200
	Pretrain	Closed
	Learning rate	0.01
	Input images size	640 × 640
	Momentum	0.937
	Weight_decay	0.0005
	Warmup_epochs	3
	Data augmentation	mosaic
Experimental environment parameters	CPU	i7-12700F
	GPU	NVIDIA GeForce RTX 3060 Ti
	GPU memory size	16 GB
	Programming language	Python-3.11.5
	Operation system	Win 11
	Module platform	Pytorch-2.0.0 + cuda11.8

**Table 2:** Performance comparison of the improved backbone model.

Model	mAP@0.5(%)	mAP@0.5:0.95(%)	GFLOPs
YOLOv8n	82.9	45	8.1
YOLOv8n + 1C2f_MLCA	85.1	46.5	8.1
YOLOv8n + 2C2f_MLCA	84.2	45.7	8.1
YOLOv8n + 3C2f_MLCA	84.4	46	8.1
YOLOv8n + 4C2f_MLCA	84.5	46.2	8.2

**Table 3:** Performance comparison of the improved neck model.

Model	mAP@0.5(%)	mAP@0.5:0.95(%)	GFLOPs
YOLOv8n	82.9	45	8.1
YOLOv8n + Smallerhead	86.5	46.9	12.2
YOLOv8n + Smallerhead + SENetV2	87.1	47.1	12.3

**Table 4:** Performance comparison of the improved loss function.

Model	mAP@0.5(%)	mAP@0.5:0.95(%)
YOLOv8n + CIoU	82.9	45
YOLOv8n + DIoU	78.3	36.4
YOLOv8n + WIoU	83.8	45.5
YOLOv8n+ WIoUv2	79.6	37.5
YOLOv8n+ WIoUv3	79.4	36.5
YOLOv8n + ShapeIoU	83.6	45.7
YOLOv8n + SIoU	84.1	46

**Table 5:** Ablation experiment results.

Experiment	C2f_MLCA	Smallerhead	C2f_SENetV2	SIoU	mAP@0.5	mAP@0.5:0.95	GFLOPs
1	-	-	-	-	82.9	45	8.1
2	√	-	-	-	85.1	46.5	8.1
3	-	√	-	-	86.5	46.9	12.2
4	-	-	√	-	83.7	45.6	8.1
5	-	-	-	√	84.1	46	8.1
6	√	√	-	-	86.8	46.9	12.2
7	√	-	√	-	85.3	45.6	8.1
8	√	-	-	√	84.5	45.9	8.1
9	-	√	√	-	87.1	47.1	12.3
10	-	√	-	√	87.0	46.9	12.2
11	-	-	√	√	84.4	46.3	8.1
12	√	√	√	-	87.6	47.3	12.3
13	√	√	-	√	87.3	47.2	12.2
14	√	-	√	√	85.8	45.7	8.1
15	-	√	√	√	87.5	47.3	12.2
16	√	√	√	√	87.9	47.6	12.3

**Table 6:** Detection performance comparison of different models.

Model	mAP@0.5(%)	mAP@0.5:0.95(%)	GFLOPs	Size	F1(%)
YOLOv5s	82.5	44	7.1	5 M	80
YOLOv6	82.1	43.4	11.8	8.3 M	81
YOLOv7	64.2	28.4	105.1	71.3 M	66
YOLOv7-tiny	76.1	34.4	13.2	11.7 M	76
YOLOv8n	82.9	45	8.1	6 M	81
YOLOv9c	87.6	47.4	102.3	49.2 M	84
YOLOv8-MSS	87.9	47.6	12.3	5.8 M	85

**Table 7:** Comparison of other algorithms.

Model	mAP@0.5(%)	mAP@0.5:0.95(%)	Size
Faster R-CNN	56.2	31.7	314 M
SSD	78	40.3	60.3 M
Rt-DETR-R50	70.6	39.8	86 M
YOLO-Float	83.3	42	91.8 M
YOLOv7-CA Dynamic	81.1	38.1	51.5 M
YOLOv8-MSS	87.9	47.6	5.8 M

**Table 8:** Comparison On WSODD (%).

Method	Mast	Bridge	Tree	Ship	Animal	Boat	Harbor	Platform	Person	Rock	Rubbish	Ball	Buoy	Glass	mAP@50
Basic	77.6	98.2	90.4	91	89.3	89.4	93.1	77	60.5	72	60	62.4	71.3	53	77.5
Ours	79.2	97.4	92.5	91.8	97.3	90.3	92.5	78.1	71.2	72.9	63	71.1	79.9	60.8	80.6

**Table 9:** Comparison on Visdrone2019 (%).

Method	Pedestrian	People	Bicycle	Car	Van	Truck	Tricycle	Awning-t	Bus	Motor Vehicle	mAP@50
YOLOv8n	35.8	27.6	24.1	76.1	38.7	29.1	22.2	11.4	47.4	37.5	35
Ours	44	35.5	26.5	80.6	42.1	30.5	25.6	15.3	47.7	44.1	39.2

## Data Availability

All data are contained within the article. To request the data and code, please send an email to the first or corresponding author.
